# PHL5/412: On-line Medication Information for Patients: The Pharmacy Infobutton

**DOI:** 10.2196/jmir.1.suppl1.e86

**Published:** 1999-09-19

**Authors:** P Doupi, JJ Cimino

**Affiliations:** ^1^Erasmus Universiteit, RotterdamThe Netherlands

**Keywords:** Internet, Drug Information Services, Patient Education, Clinical Pharmacy Information Systems

## Abstract

**Introduction:**

Several strategies and techniques have been explored for providing health care professionals with automated access to information resources. Insufficient evidence is available though as to how this issue should be approached from the side of patients. Problems already identified in this respect are the verification of information quality and the navigation to appropriate sources. The "infobutton" application developed at Columbia University attempts to provide a comprehensive solution to both. In this paper we present the strategy employed in the development of a pharmacy infobutton offering patients "one touch access" to medication-related information available on public web sites.

**Methods:**

The development of an information button involves four consecutive phases. 1) Identifying the users' questions Users' likely questions were elicited from several complementary sources: a) publications regarding information demands on traditional drug information resources b) users' questions submitted to web sites covering the topic of medications c) information gaps as they are offset by the commonest medication misuses d) recommendations of regulatory authorities for the provision of medication information to the public. Subsequently we used the identified topics to construct generic question templates which are utilised in query formulation. 2) Identifying the appropriate information source Sites were identified and reviewed with respect to quality of content, using predefined evaluation checklists. Consequently a database was created containing URLs of pertinent HTML documents and indexing information. 3) Composing a retrieval strategy. All medications prescribed at Columbia-Presbyterian are represented in coded form in the Medical Entities Dictionary (MED). Queries to the URL database can be constructed using trade/generic names or ingredient terms taken from the MED, in combination with generic question templates. 4) Presenting the results to the user. The Patient Clinical Information System (PatCIS) places an infobutton icon next to each drug name. When users select an infobutton they are presented with a list of possible questions of interest. Each question is a link to one or more resources, using medication-specific queries.

**Results:**

Information buttons linked to Medline and Micromedex are presently available for all medications in the MED. Those providing access to Internet resources will be offered for a restricted number of medications, in an initial pilot phase. Recruitment of patients is under way for a first round of identifying the application's usefulness and shortcomings.

**Discussion:**

The role of the Internet in the process of health care delivery continues to grow, increasing the demand for applications that make efficient use of the medium's capabilities. The PatCIS "infobuttons" are such an application offering an innovative approach to the challenge of enhancing public awareness of health issues.

